# Dental Ethics

**Published:** 1890-08

**Authors:** J. Wardhouse

**Affiliations:** Grand Rapids, Mich.


### Dental Ethics—From the Standpoint of a Young Practitioner.

BY DR. J. WARDHOUSE, GRAND RAPIDS, MICH.

It is with a feeling of timidity that I come before you to present a paper on a subject which has been in the past, and is to-day occupying a place foremost in our dental literature.

Timidity, because being a young practitioner myself, it is natural that I should feel incapable of profitably addressing the older members of the profession.

Since my subject is discussed from the standpoint of a young practitioner, however, the paper could not be presented consistently by one old in the profession. And, moreover, it is possible that a brief consideration of the subject from this standpoint may not be wholly without value to the older practitioners.

Ethics defined in its broadest sense is the science of human duty, including a body of rules drawn from this science. It is applied as well to a body of rules of practice in respect to a single class of human actions, such as social, political, or dental ethics. From these general statements, then, we are able to formulate a definition of the subject before us.

DENTAL ETHICS is a body of rules (relating to dental practice and that pertaining to it) drawn from the science of human duty.

The task before us now is to formulate this body of rules from our point of view, and in so doing we hope to provoke a discussion out of which a better code may come. Even though the discussion be sufficiently heated to reduce our paper to ashes, we shall not regret it, for out of its ashes we trust that, phœnix-like, there shall rise that which is far more enduring.

THE ETHICAL RELATION OF PRACTITIONER TO PATIENT.—Although it is our intention to keep clearly defined the distinction between our subject and the legal aspect, we deem it well to give a brief summary of the legal side as discussed most ably in one of our leading journals.*

"A dentist, like any one else, who offers his services to the public, generally for employment in a professional capacity, contracts with his employer; first, that he possesses a reasonable and ordinary degree of skill and learning; and second, that he will use a reasonable and ordinary degree of care and diligence in the exercise of his skill and in the application of his learning to accomplish the purpose for which he is employed."

And further it is stated that, "Where the result desired, as the cure in the case before us, depends both upon the skill in the use of means and the influences of other causes, the law raises no such implied engagement; it regards the undertaking to be only

*Daniel Nason in Odontographic Journal, October, 1887.

for the use of proper means. The retainer of a lawyer obliges him to the right conduct of the suit, but not for the judgment of the court, for that is beyond his control. The retainer of a physician obliges him to the employment of ordinary medical skill in the treatment of the patient; the cure is not with him. The surgeon called to attend a patient with a broken or dislocated limb impliedly engages the ordinary skill of his profession adjusting it; he is not supposed to engage to cure or to insure a recovery."

Moreover do we find clearly brought out from the discussions of judges and juries in cases cited, the fact that, first, the dentist is not allowed to use the most approved means, or be skilled in the more difficult applications of the advanced research in the profession. The law holds him simply to the lowest degree of knowledge that will admit one to practice; secondly, that any negligence on the part of the patient is sufficient to counteract the lack of skill on the part of the practitioner: and lastly, that in no case is the practitioner required to make use of especial adaptation of skill on his own part or that of others.

Here we have the legal side of the case and we shall venture to treat the ethical side as beginning where the legal ends.

Let us assert first of all that the young practitioner who starts out with his aim no higher than that required to satisfy the law, will soon prove himself of but little value to the profession and the profession of but little value to him.

The ethical relation of practitioner to patient is based upon the determination of the practitioner to render to the patient full value of honest, conscientious service for the compensation asked. If he has been conscientious from the beginning he will have first asked himself the question: "Am I so constituted and have I such ability that I can take up this profession and render valuable service to my patients?" This point once settled he should further conclude that, "It is my next duty to seek such instruction as will develop such ability most perfectly." Now, located in his office, let him keep ever in mind his duty to those he proposes to serve, rather than the idea that each blow with the mallet will place so many cents to his credit. He should remember too, that the "golden rule" is as valuable in the ethical treatment of his patient as the "golden foil" in the dental treatment. We will now assume that every young practitioner has carried out what we have laid down for him, but that he has also a strong desire to make known his good works to the world. Here let me insist on the fact that his good works are much nearer the brains of the people when put into their mouths than when put in the newspapers. "The best advertisement is a pleased patron."

Ethical Relation of Practitioner to Practitioner.—Upon this subject there is no legal side to state. The law is unwritten other than the relation of man to man. But on the ethical side there is much that I feel should be written, if not elsewhere, upon the tablets of our memories.

Let me introduce the first point that I wish to make under this head with an illustration. In a certain prosperous city of a population of one hundred thousand inhabitants, we will assume there are twenty dentists in active practice. In the morning paper the dentist read the card of J. M. Jones, D.D.S., office at No. 140, "Pleased to have you call" sheet. Dr. Smith, one of the most prominent of the twenty dentists mentioned above, on going to his office that morning drives out of his way sufficiently to pass Dr. Jones' office, drops in, finds a bright young man with an office of a type to commend itself to patients and fellow practitioners, and the young doctor in keeping with his office. Dr. Smith learns that Dr. Jones is a graduate of the very reputable college of G——. After a social call he leaves for his office. During the month that follows he learns from the Dean of the College of G——that the young dentist in question is in every way worthy of a hearty reception into the profession. During the following years that the young practitioner must learn to wait as well as labor, Dr. Smith shows to him every social and professional courtesy that is possible to a fellow worker. What can we say of the remaining nineteen members of the profession; with equal effort do they extend to our young practitioner the conventional "cold shoulder." Has Dr. Smith the correct ethics in the case, or have the select nineteen? This question I need not answer for you.

Let me illustrate again. Mrs. X, a patient of Dr. A, enters the office of Dr. B. "Good morning Dr. B; Dr. A has done my work for some time, but the last filling he put in dropped right out in just a little while, and his other work begins to hurt me and I don't think it good, and he is getting so that he charges ever so much when he does just a little work, and Mrs. Y says it is just the same way with her work, and Mrs. Z recommended that we come to you," she exclaimed all in a single breath.

"Good morning," returns Dr. B. "I fear you have not been wholly just to Dr. A, but if you will kindly take the chair I will look over your work. Now Mrs. X surely you have some very good work; I see you have lost the filling from this molar, but I have no doubt that the blame does not rest with Dr. A. The dentist is obliged to acknowledge that failure at times is possible for him, as for those in other walks of life. Dr. A is certainly entitled to the credit of having done some very good work for you."

On the same morning in another part of the city a second Mrs. X, a patient of Dr. C, enters the office of Dr. D, with sentiments similar to those of her sister across the river. Does she meet with like treatment from Dr. D? "Good morning Mrs. X," he returns. "If you will take my chair I will look over your work. Now Mrs. X," he continues, "it is a shame you should have been swindled in this manner. This work is a disgrace to the profession, and the charges are excessive."

The work I have assumed to be similar in the two cases cited. Again I ask has Dr. B or Dr. D the right ethics in the case? And again may I say, this question I need not answer for you.

I will not illustrate this part of my subject further. I wish, however, to close it with the statement that he who is governed by our "Code of Ethics," will serve the patient of the absent or ill fellow practitioner without seeking to steal his patient from him.

Ethical Relation of Practitioner to the Profession.—It will be disputed by none, we think, that of all the professions there is not one for which more rapid progress can be claimed than for the one we represent. The question naturally arises, To whom is the credit for this progress due? I venture with boldness to assert that a code of ethics not far different from that we have endeavored to present in our imperfect way to you, has been no small factor in the problem. From whence comes progress in any profession or vocation in life? I answer without fear of contradiction, that it comes only through conscientious, earnest toil, through a sacrifice of time and money on the part of him who is endowed not only with ability, but also with a love for the cause he represents that transcends the love of mere personal gain.

From the standpoint of the young practitioner I declare most emphatically that my love for the profession and my faith in the code of ethics that has governed our association, is such that I prefer to take my stand with the practitioner who seeks to advertise his works by means of patient labor rather than with the practitioner who seeks as his means the advertising in our daily papers—objectionable cuts showing the agony suffered by the prevalent method and the pleasure resulting from the advertiser's way of operating, and which, if harbored by us would result in a few years to the profession resorting to more disgusting means—may be like this: The dentist would have photos taken of himself and advertise "One of these beautiful chromos given away with every gold filling." In conclusion let me say, that as intelligent men, men having the interest of the profession at heart, should we allow ourselves to be brought to the level of the advertiser, or should we with greater earnestness in our work try to bring them to our standard? I think we will all agree on the latter. How can this be best accomplished? Let me offer a few suggestions.

In the first place let our dental schools and colleges and examining boards be more particular and set the example. Let the younger members of the profession, if qualified, receive the endorsement and good wishes of the older members. Let there be more familiarity among the dentists. Let each one show by word and action fidelity to our Code of Ethics.

And lastly, let each one strive to make our annual meetings a success, and our local societies more numerous.

